# Atrial natriuretic peptide attenuates metabolic acidosis and inflammation of the kidney, lung and heart in a rat model of renal ischemia-reperfusion injury

**DOI:** 10.1186/cc12350

**Published:** 2013-03-19

**Authors:** M Khin Hnin Si, C Mitaka, M Tulafu, S Abe, S Ikeda

**Affiliations:** 1 Tokyo Medical and Dental University Graduate School, Tokyo, Japan

## Introduction

Renal ischemia-reperfusion injury (IRI) is a common cause of acute kidney injury and occurs in various clinical conditions including shock and cardiovascular surgery. Renal IRI releases proinflammatory cytokines within the kidney. Atrial natriuretic peptide (ANP) has natriuretic, diuretic and anti-inflammatory effects [1] and plays an important role of regulating blood pressure and volume homeostasis. The hypothesis was that renal IRI induces inflammation not only in the kidney but also in remote organs such as the lung and heart and ANP attenuates renal injury and inflammation in the kidney, lung and heart.

## Methods

Male Sprague-Dawley rats were anesthetized with pentobarbital. Tracheostomy was performed and rats were ventilated at VT 10 ml/kg with 5 cmH_2_O PEEP. The right carotid artery was catheterized for blood sampling and continuous blood pressure measurements. The right femoral vein was catheterized for infusion of saline or ANP. Rats were divided into three groups; IRI group (*n *= 10), left renal pedicle was clamped for 30 minutes; IRI+ANP group (*n *= 10), left renal pedicle was clamped for 30 minutes, ANP (0.2 μg/kg/minute, for 3 hours 25 minutes) was started 5 minutes after clamp; and Sham group (*n *= 6), the sham-operated rats. Hemodynamics, arterial blood gas, and plasma lactate levels were measured at baseline and at 1 hour, 2 hours and 3 hours after declamp. The mRNA expression of IL-6 in the kidney, lung, and heart were measured. The kidney, lung and heart were immunostained to examine the localization of IL-6 and NF- B and assigned an expression score. The wet/dry ratio of the lung was also measured.

## Results

Renal IRI induced metabolic acidosis, pulmonary edema, mRNA expression of IL-6 in the kidney, lung and heart. Renal IRI increased immunohistochemical localization of IL-6 in the proximal convoluted tubule of the left kidney and NF-KB in the bronchial epithelial cells of the lung. ANP attenuated metabolic acidosis, pulmonary edema and expression of IL-6 mRNA in the kidney, heart, and lung. ANP decreased immunohistochemical localization of IL-6 in the left kidney and NF-KB in the lung.

## Conclusion

These findings suggested that inflammation within the kidney after renal IRI was extended into the lung and heart. ANP attenuated metabolic acidosis and inflammation in the kidney, lung and heart in a rat model of renal IRI. ANP may attenuate organ crosstalk between the kidney, lung and heart.
